# Ubiquitination of Lysine 867 of the Human SETDB1 Protein Upregulates Its Histone H3 Lysine 9 (H3K9) Methyltransferase Activity

**DOI:** 10.1371/journal.pone.0165766

**Published:** 2016-10-31

**Authors:** Kenji Ishimoto, Natsuko Kawamata, Yoshie Uchihara, Moeka Okubo, Reiko Fujimoto, Eiko Gotoh, Keisuke Kakinouchi, Eiichi Mizohata, Nobumasa Hino, Yoshiaki Okada, Yasuhiro Mochizuki, Toshiya Tanaka, Takao Hamakubo, Juro Sakai, Tatsuhiko Kodama, Tsuyoshi Inoue, Keisuke Tachibana, Takefumi Doi

**Affiliations:** 1 Laboratory of Molecular Medicine, Graduate School of Pharmaceutical Sciences, Osaka University, Suita, Osaka, Japan; 2 Laboratory for System Biology and Medicine, Research Center for Advanced Science and Technology, The University of Tokyo, Meguro, Tokyo, Japan; 3 Department of Applied Chemistry, Graduate School of Engineering, Osaka University, Suita, Osaka, Japan; 4 Department of Quantitative Biology and Medicine, Research Center for Advanced Science and Technology, The University of Tokyo, Meguro, Tokyo, Japan; 5 Division of Metabolic Medicine, Research Center for Advanced Science and Technology, The University of Tokyo, Meguro, Tokyo, Japan; Russian Academy of Medical Sciences, RUSSIAN FEDERATION

## Abstract

Posttranslational modifications (PTMs) of proteins play a crucial role in regulating protein-protein interactions, enzyme activity, subcellular localization, and stability of the protein. SET domain, bifurcated 1 (SETDB1) is a histone methyltransferase that regulates the methylation of histone H3 on lysine 9 (H3K9), gene silencing, and transcriptional repression. The C-terminal region of SETDB1 is a key site for PTMs, and is essential for its enzyme activity in mammalian and insect cells. In this study, we aimed to evaluate more precisely the effect of PTMs on the H3K9 methyltransferase activity of SETDB1. Using mass spectrometry analysis, we show that the C-terminal region of human SETDB1 purified from insect cells is ubiquitinated. We also demonstrate that the ubiquitination of lysine 867 of the human SETDB1 is necessary for full H3K9 methyltransferase activity in mammalian cells. Finally, we show that SETDB1 ubiquitination regulates the expression of its target gene, serpin peptidase inhibitor, clade E, member 1 (*SERPINE1*) by methylating H3K9. These results suggest that the ubiquitination of SETDB1 at lysine 867 controls the expression of its target gene by activating its H3K9 methyltransferase activity.

## Introduction

Posttranslational modifications (PTMs) of proteins play a crucial role in regulating protein-protein interactions, enzyme activity, subcellular localization, and stability of the protein [1, 2]. These PTMs include phosphorylation, glycosylation, ubiquitination, sumoylation, acetylation, methylation, and lipidation. Recent studies have provided evidence that the PTMs of histone methyltransferases control their enzyme activity and biological functions. Enhancer of zeste, drosophila, homolog 2 (EZH2), which is a histone-lysine N-methyltransferase enzyme, is modified via O-GlcNAcylation by O-linked *N*-acetylglucosamine transferase [3]. O-GlcNAcylation of EZH2 is known to upregulate the stability of EZH2 and increase cellular histone H3 lysine 27 trimethylation level. Sulforaphane-induced PTMs (acetylation and ubiquitination) of suppressor of variegation 3–9, drosophila, homolog 1 (SUV39H1) decreases histone H3 lysine 9 (H3K9) trimethylation, leading to enhanced apoptotic signaling in metastatic prostate cancer cells [4]. H3K9 methyltransferase G9a is a self-methylating lysine methyltransferase that mediates the in vivo interaction with the epigenetic regulator heterochromatin protein 1 [5]. Hence, studying PTMs of enzymes is a useful way of understanding novel mechanisms by which their activity is regulated.

SET domain, bifurcated 1 (SETDB1) is a histone methyltransferase that regulates the methylation of histone H3 on lysine 9, gene silencing, and transcriptional repression [6–8]. Increased expression of SETDB1 has been observed in melanoma, lung cancer, and liver cancer [9–11]. Its overexpression has been shown to promote cell growth, invasiveness, and tumorigenesis. It is also involved in the epigenetic regulation of gene expression by Notch signaling in colorectal cancer [12]. SETDB1, along with Krüppel-associated box-associated protein 1, is involved in H3K9 trimethylation which results in the silencing of endogenous and introduced retroviruses in mouse embryonic stem (ES) cells [13]. SETDB1 is also a critical regulator of 3T3-L1 adipocyte differentiation [14]. Thus, SETDB1 controls a variety of intracellular physiologic processes through its enzyme activity.

**Fig 1:**
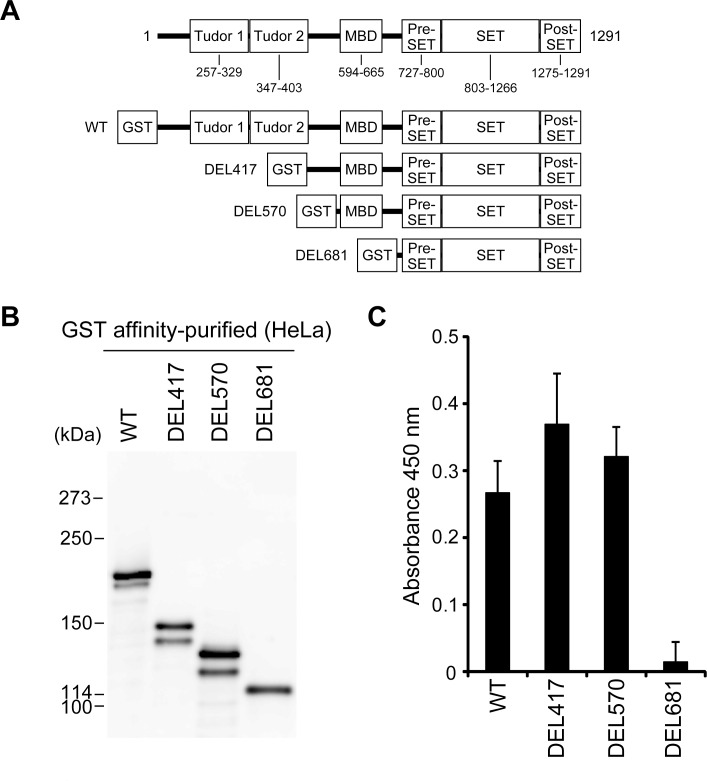
The PTMs of SETDB1 are associated with its H3K9 methyltransferase activity in HeLa cells. (A) Schematic representation of the domain structure of human SETDB1 and the deletion mutants. Amino acid sequence is numbered in accordance with the UniProt numbering scheme; the tandem Tudor domains, methyl-CpG-binding domain (MBD), pre-SET domain, SET domain, and post-SET domain are indicated. (B) SETDB1 proteins were expressed as GST fusion protein in HeLa cells and purified on glutathione-sepharose beads. The purified SETDB1 proteins were resolved on 5% SDS-PAGE, and electroblotted onto PVDF membrane. Western blot analysis of GST-SETDB1 proteins was probed with anti-GST antibody. (C) H3K9 methyltransferase activity of GST affinity-purified SETDB1 proteins in HeLa cells was measured. The values represent means±SEM (n = 3).

The human SETDB1 protein (1291 amino acids, 143 kDa) is organized into six domains (Fig 1A). The tandem Tudor domains in the N-terminal region are involved in protein-protein interactions [15, 16]. The methyl-CpG-binding domain (MBD) in the middle region is thought to mediate methyl-CpG binding and protein-protein interactions [7]. The C-terminal region with the pre-SET, SET, and post-SET domains, as found in most SET family members, is responsible for H3K9 specific lysine methylation [6, 7]. Interestingly, while it is understood that SETDB1 needs to undergo posttranslational modification to function as an H3K9 methyltransferase [7], the precise mechanisms that regulate the PTMs of SETDB1 are yet to be fully elucidated. In this study, we aimed to evaluate more precisely the effect of PTMs on the H3K9 methyltransferase activity of SETDB1. Using mass spectrometry analysis, we also examined the type of PTMs SETDB1 undergoes. Furthermore, we investigated whether the PTMs of SETDB1 play a role in regulating the expression of its target gene, serpin peptidase inhibitor, clade E, member 1 (*SERPINE1*).

## Materials and Methods

### Plasmid Constructs

The human SETDB1 expression vector (pDEST27–hSETDB1 or pcDNA3-hSETDB1) was created by cloning the appropriate cDNA from HEK293 cells into the pDEST27 vector or the pcDNA3 vector [17]. The baculovirus SETDB1 expression vector was transferred to pDEST20 by recombination using the Gateway cloning system (Life Technologies) to generate the plasmid pDEST20-hSETDB1. A deletion construct was made and site-directed mutations were introduced into SETDB1 by PCR.

### Cell Culture

HeLa cells were cultured in DMEM supplemented with 10% heat-inactivated FBS, 100 IU/ml penicillin, and 100 μg/ml streptomycin. Sf9 cells were cultured in Grace's supplemented media (Life Technologies) containing 10% FCS, 0.1% Pluronic F-68 (Life Technologies), 100 IU/ml penicillin, and 100 μg/ml streptomycin (P/S) in a 1 l spinner flask at 27°C.

### Affinity Purification of GST-tagged Proteins

DNA transfection of HeLa cells was performed with Lipofectamine 2000 (Invitrogen), as described previously [18]. One day before transfection, the cells were seeded at a density of 7×10^5^ cells on 6 cm dishes. The cells were transfected with 8 μg of the GST-SETDB1 expression vector. Five hours after transfection, the cells were cultured in DMEM supplemented with 10% FBS and 2% P/S. The cells were harvested 24 h after transfection, and lysed in 25 mM Tris-HCl (pH 7.4) containing 150 mM KCl, 5 mM EDTA, 1% NP-40, 0.2% sodium deoxycholate, 1 mM DTT, 2.5 mM sodium pyrophosphate, 10 mM β-glycerophosphate, 10 mM sodium fluoride, and protease inhibitor cocktail (Sigma). The cell extract was added to glutathione-sepharose 4B (GE Healthcare) and the mixture was incubated with rotation at 4°C for 90 min. The beads were collected by centrifugation, rinsed three times, and the bound GST-SETDB1 was detected by SDS-PAGE and immunoblotting.

Sf9 cells were harvested 72 h postinfection. The cell pellet was suspended in lysis buffer [30 mM Tris-HCl (pH 8.0), 150 mM NaCl, 5 mM DTT, 1 mM phenylmethylsulfonyl fluoride, 50 μM leupeptin, 1 μg/ml pepstatin A, 2.5 mM sodium pyrophosphate, 10 mM β-glycerophosphate, and 50 mM sodium fluoride], and the cells were lysed by sonication. GST-SETDB1 proteins from cell extracts were purified using glutathione-sepharose 4B.

### Mass Spectrometry Analysis

Samples were sent to the Center for Medical Research and Education, Osaka University Graduate School where they were analyzed by mass spectrometry as a custom contract service. To prepare the sample for mass spectrometry analysis, GST affinity-purified DEL570 [GST-SETDB1 (570–1291), (Fig 1A)] was cleaved with PreScission protease. The cleaved protein was purified on HiTrap Q HP columns (GE Healthcare) and on Superdex 200 (GE Healthcare) gel filtration column. The purified SETDB1 (570–1291) was separated via 4–12% SDS-PAGE with subsequent visualization of proteins by fast SimplyBlue SafeStain (Life Technologies). The stained gel bands were cut and subjected to tryptic in-gel digestion. Digested peptides were separated by nano-LC (Ultimate 3000, Thermo Scientific) and analyzed by MS/MS (Q Exactive, Thermo Scientific). The resulting spectra were examined using the Mascot Software (Matrix Science).

### Preparation of Cell Lysates

To prepare whole cell extracts, HeLa cells were lysed in 25 mM Tris-HCl (pH 7.5), 150 mM NaCl, 5 mM EDTA, 1% NP-40, 1% sodium deoxycholate, 0.1% SDS, and protease inhibitor cocktail (Sigma).

### Immunoblot Analysis

Each cell extract was resolved on SDS-PAGE and electroblotted onto the PVDF membrane. Western blot analysis was performed using anti-SETDB1 antibody (Bethyl Laboratories), anti-GST antibody (Santa Cruz Biotechnology), anti-mono- and polyubiquitin (FK2) antibody (Enzo Life Sciences), and anti-lamin B2 antibody (Abcam). The signals were visualized with the SuperSignal West Dura extended duration chemiluminescent substrate (Pierce).

### Measurement of H3K9 Methyltransferase Activity

The GST-SETDB1 proteins were purified by affinity chromatography on glutathione-sepharose 4B. Measurement of the H3K9 methyltransferase activity was carried out using the EpiQuik Histone Methyltransferase Activity/Inhibition Kit (H3K9), as recommended by the manufacturer (EPIGENTEK). Absorbance at 450 nm was measured by a Microplate reader Model 550 (Bio-Rad).

### RNA Extraction and Real-Time RT-PCR Analysis

DNA transfection of HeLa cells was performed with Lipofectamine 2000 (Invitrogen), as described previously [18]. The expression vector was transfected into HeLa cells twice with 48 h intervals between transfection. One day before transfection, the cells were seeded at a density of 2.5 × 10^5^ cells on 6-well plates. The cells were transfected with 2.8 μg of the expression vector. Forty-eight hours after the first transfection, the cells were transfected with 2.8 μg of the expression vector again. Twenty-four hours after the second transfection, the cells were cultured in DMEM supplemented with 10% FBS and 2% P/S. The cells were collected 120 h after the first transfection. Total RNA extraction and cDNA synthesis were performed as described previously [19]. PCRs were performed with a QuantiTect® SYBR Green PCR Kit (Qiagen). Amplification specificity was verified by visualizing the PCR products on an ethidium bromide-stained 2% agarose gel. Cyclophilin A mRNA was used to normalize each expression datum. The primers used were: 5′-CCCAGGATCTGCATAAAGGA-3′ and 5′-TCAGCAGGAGGGTGGTAATC-3′ for SETDB1; 5′-GACTTCACGAGTCTTTCAGACCAA-3′ and 5′-ACTATGACAGCTGTGGATGAGGAG-3′ for SERPINE1; 5′-GACTTTTCCCGAAGGAGGAACT-3′ and 5′-GCTGCAGTTGAAAGGCTTCT-3′ for kruppel-like factor 11 (KLF11); 5′-GTGAGGAGGCAAGGTTCTCAGG-3′ and 5′-GATCTTCTCCTTTGGTGCTCCTCTA-3′ for melanoma antigen, family A, 5 (MAGEA5); 5′-GGACCTTTTCTTCAGAGGGTGA-3′ and 5′-CTGTCTCCTCAGAACCTGGATGC-3′ for melanoma antigen, family A, 12 (MAGEA12); and 5′-GCGTCTCCTTTGAGCTGTTT-3′ and 5′-TCACCACCCTGACACATAAACC-3′ for cyclophilin A.

### Chromatin Immunoprecipitation (ChIP) Assay

ChIP assays were performed as described previously [20]. DNA transfection of HeLa cells was performed as described above. At 120 h after the first transfection, cells were crosslinked with 1% formaldehyde for 10 min at room temperature. Fixation was completed following the addition of glycine with a final concentration of 200 mM. The crosslinked genomic DNA was sheared by sonication using a Digital Sonifier Model 250 (Branson). The DNA-protein complexes were immunoprecipitated with Dynabeads Protein G (Invitrogen). An antibody directed against Histone H3 (tri methyl K9) (Abcam) was used. The precipitated DNA was analyzed by real-time PCR using the primers 5'-GTTCGCCAAAGGAAAAGCAGG-3' and 5'-GTGTCTGTCTCTCCCGGATGTC-3', which target a region located upstream of the transcription start site of the SERPINE1 promoter, or primers 5'-GCCCCTGTTTACGGAGCATTTC-3' and 5'-TGCGTGATACTGGGCTAGGAAC-3' which target the genomic region (ch10:79154928–79155075).

### Statistical Analysis

All data are presented as mean±standard errors (SEM). Statistical analyses were performed using the unpaired Dunnett's test with Statcel version 3.0 (OMS Publishing Inc.).

## Results

### PTMs of the C-terminal Region of SETDB1 are Necessary for the Activation of H3K9 Methyltransferase

Shultz et al. reported that SETDB1 purified from the cell extracts was observed as two bands by SDS-PAGE analysis and Coomassie blue staining [7]. We hypothesized that the upper band of SETDB1 in the SDS-PAGE analysis is because of PTMs and that the PTMs of SETDB1 are involved in its H3K9 methyltransferase activity (see below and discussion).

First, to reveal the amino acid site and type of PTMs in SETDB1, we prepared the affinity-purified GST fusion SETDB1 full-length protein (WT) from transfected HeLa cell extracts (Fig 1A). We also prepared GST fusion proteins from the three types of N-terminal deletion mutants of SETDB1: GST-SETDB1 (417–1291) (DEL417), GST-SETDB1 (570–1291) (DEL570), and GST-SETDB1 (681–1291) (DEL681). The affinity-purified GST fusion proteins from HeLa cells were subjected to immunoblot analysis with an anti-GST antibody (Fig 1B). WT was observed as two closely located bands with a molecular weight of about 200 kDa. Similar pairs of bands were seen for DEL417 and DEL570, whereas DEL681 was observed as a single band at about 114 kDa. These results indicate that the region between the amino acids 570 and 1291 that contains the MBD, pre-SET, SET, and post-SET domains is indispensable for the PTMs of SETDB1 in HeLa cells.

Next, we investigated the H3K9 methyltransferase activity using the four kinds of purified SETDB1 proteins from HeLa cells (Fig 1C). WT, DEL417, and DEL570 exhibited distinct methyltransferase activity, whereas it was found to be impaired in DEL681. These results suggest that the C-terminal region of SETDB1 (from amino acids 570 to 1291) is necessary for its methyltransferase activity.

We also expressed and affinity-purified SETDB1 WT and the three mutants from baculovirus-infected Sf9 cell extracts, and performed the immunoblot analysis and H3K9 methyltransferase activity assay. The results obtained were similar to that with affinity-purified SETDB1 from HeLa cell extracts (S1 Fig). These results suggest that the C-terminal region of SETDB1 (570–1291) plays a key role in its PTMs, and is essential for its enzyme activity in mammalian and insect cells.

### SETDB1 Undergoes Ubiquitination

**Fig 2:**
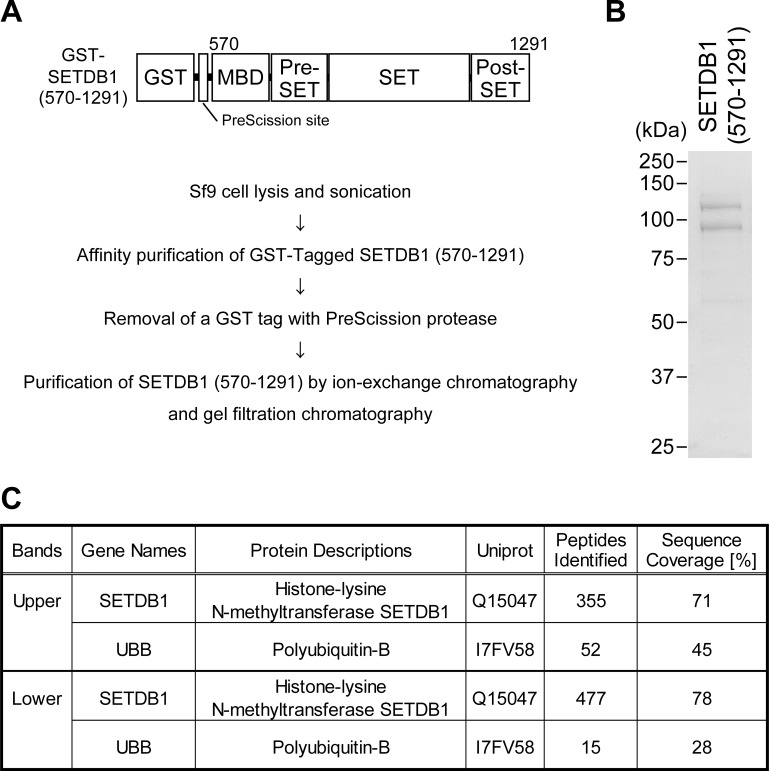
GST-SETDB1 (570–1291) protein expressed in Sf9 cells was prepared using various types of chromatography for mass spectrometry analysis. (A) Schematic representation of the construction of GST-SETDB1 (570–1291) (top) and the flow chart for SETDB1 (570–1291) purification (bottom). (B) SETDB1 (570–1291) purified using chromatography was separated by SDS-PAGE and analyzed by Coomassie blue staining. (C) Summary of the mass spectrometry analysis data, showing the comparison between the upper and lower band of SETDB1 (570–1291).

The pattern of PTMs of SETDB1 (570–1291) purified from HeLa cells did not differ from that of the protein purified from Sf9 cells. To identify the type of PTMs in SETDB1 using mass spectrometry, we performed a large-scale preparation and purification of GST-SETDB1 (570–1291) using Sf9 cells. GST-SETDB1 (570–1291) from Sf9 cell extracts was affinity-purified by using glutathione-sepharose 4B, and GST was removed from GST-SETDB1 (570–1291) by enzymatic cleavage (Fig 2A). Subsequently, SETDB1 (570–1291) was purified by using ion-exchange chromatography, and gel filtration chromatography. The two bands derived from SETDB1 (570–1291) were separated by SDS-PAGE, and clearly visualized by Coomassie blue staining (Fig 2B). This was followed by in-gel digestion of these bands, and liquid chromatography-tandem mass spectrometry (LC-MS/MS) analysis for protein identification (Fig 2C and S1 Table). The upper band revealed that the sequences of all the peptides coincided with those expected from the sequence of SETDB1 (570–1291) (71% sequence coverage). The lower band was also identified as SETDB1 (570–1291) fragments (78% sequence coverage). Although PTMs such as phosphorylation were found in both bands, PTMs specific to the upper band were not found (S1 Table). However, we found polyubiquitin-B (UBB) peptides in each of the two bands. Fifty-two peptides identified in the upper band covered 45% of the UBB amino acid sequence, while fifteen peptides identified in the lower band covered 28% of the UBB amino acid sequence. These results suggest that the type of PTM in higher molecular weight SETDB1 is ubiquitination.

**Fig 3:**
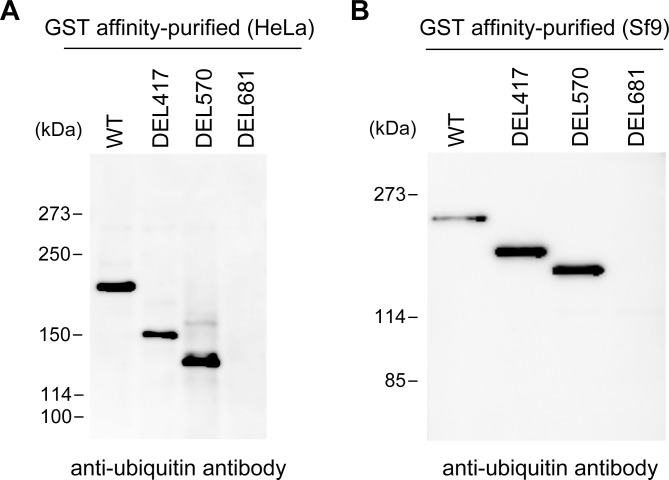
The region between amino acids 570 and 680 may contain the ubiquitination site in SETDB1. (A) GST affinity-purified SETDB1 proteins in HeLa cells were resolved on 5% SDS-PAGE. (B) GST affinity-purified SETDB1 proteins in Sf9 cells were resolved on 7% SDS-PAGE. Western blot analyses were performed using anti-ubiquitin antibody.

To ascertain whether ubiquitin is attached to SETDB1, we performed immunoblot analysis with an anti-ubiquitin antibody using the four kinds of affinity-purified SETDB1 proteins from HeLa cell extracts. We were able to detect a single band of ubiquitinated protein from WT, DEL417, and DEL570 (Fig 3A), although two bands were observed from each of these proteins in Fig 1B. On the other hand, we were not able to detect ubiquitin in the DEL681 protein. We also performed the immunoblot analysis using the purified SETDB1 proteins from Sf9 cell extracts, and the results were consistent with the results from HeLa cells described above (Fig 3B). These results reveal that the upper band of SETDB1 was modified by ubiquitin.

### SETDB1 Ubiquitination at Lysine 867 is Necessary for Full H3K9 Methyltransferase Activity

**Fig 4:**
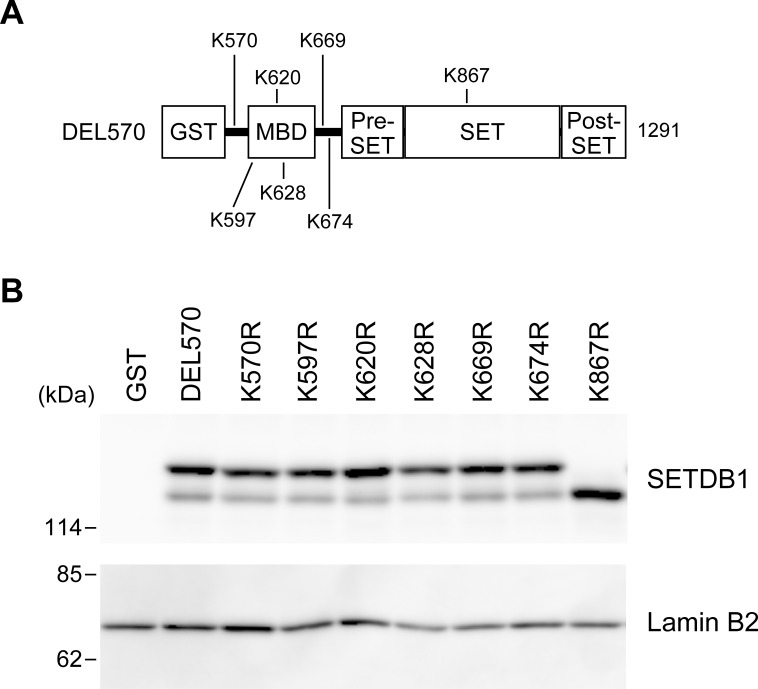
Lysine 867 located in the SET domain is the site for PTMs in HeLa cells. (A) Schematic view of the mutation map for GST-SETDB1 (570–1291). (B) Twenty-four hours after transfection, cell extracts were prepared, and the extracts were subjected to SDS-PAGE. Western blot analyses were probed with anti-SETDB1 antibody or anti-lamin B2 antibody.

Next, we examined the site of ubiquitin-modified lysine residue on SETDB1. Since DEL681 does not show evidence of ubiquitination in Fig 3, ubiquitin-modified lysine residue is inferred to be located between amino acids 570 and 680 of SETDB1. There are three lysine residues (K597, K620, and K628) within the MBD (from 594 to 665 on SETDB1) present in this region (Fig 4A). In addition, three more lysine residues (K570, K669, and K674) exist in the region outside the MBD. Therefore, we introduced a mutated version of each of these six lysine residues into the DEL570 protein and performed immunoblot analysis with an anti-SETDB1 antibody using HeLa cell extracts (Fig 4B). Unexpectedly, two bands were observed in DEL570 as well as in all the mutated constructs suggesting that they had undergone PTMs. Therefore, we inferred that the ubiquitin-modified lysine is not located between amino acids 570 and 680 on SETDB1; rather it should be located between amino acids 681 and 1291.

To identify the site of ubiquitination of SETDB1 between amino acids 681 and 1291, we reanalyzed the LC-MS/MS analysis results (S2 Fig). SETDB1 peptides corresponding to the region between amino acids 845 and 886 were detected in the lower band but not in the upper band. When ubiquitin-conjugated protein is digested with trypsin, proteolysis cannot occur at the ubiquitin-modified lysine residues [21, 22]. Therefore, if lysine 867, which is present in the region between amino acids 845 and 886, was modified by ubiquitin, it would not have been digested by the enzyme. This would have resulted in a long peptide that the mass spectrometer would not have been able to detect. We then generated a SETDB1 mutant in which lysine 867 was converted to an arginine residue (K867R), and performed immunoblot analysis in HeLa cell extracts (Fig 4B). We observed a single band in K867R, in the same location as the lower band in DEL570, whereas two bands were observed in DEL570. These results suggest that the lysine 867 is the residue necessary for PTMs of SETDB1.

**Fig 5:**
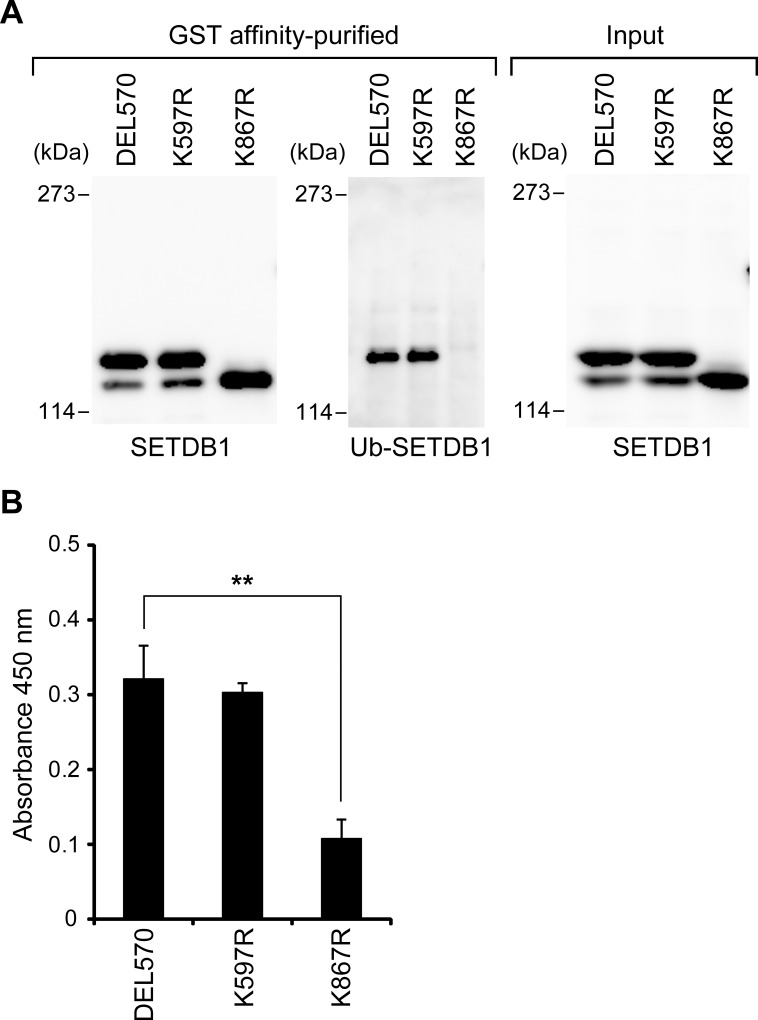
Ubiquitination of K867 of GST-SETDB1 (570–1291) upregulates its H3K9 methyltransferase activity in HeLa cells. (A) SETDB1 proteins were expressed as GST fusion proteins in HeLa cells and purified on glutathione-sepharose beads. The purified SETDB1 proteins were resolved on 5% SDS-PAGE, and electroblotted onto PVDF membranes. Western blot analyses of GST-SETDB1 proteins were probed with anti-SETDB1 antibody or anti-ubiquitin antibody. (B) H3K9 methyltransferase activity of the GST affinity-purified SETDB1 proteins in HeLa cells was measured. The values represent means±SEM (n = 3). **P<0.01 compared with the value of DEL570.

Next, we affinity-purified DEL570, K597R, and K867R from HeLa cell extracts, and performed immunoblot analysis with an anti-ubiquitin antibody (Fig 5A). In DEL570 and K597R, where two bands were observed in Fig 4B, we detected a single band of ubiquitin-modified SETDB1. In K867R where a single band was observed in the location of the lower band, the ubiquitin-modified SETDB1 was not detected. These results suggest that lysine 867 is a site for the ubiquitination of SETDB1.

We examined the effects of SETDB1 ubiquitination on the H3K9 methyltransferase activity in affinity-purified SETDB1 from HeLa cell extracts. In K597R, which is modified by ubiquitin, methyltransferase activity at the same level as DEL570 was observed (Fig 5B). On the other hand, K867R showed approximately 30% of the methyltransferase activity seen with DEL570. We also investigated the methyltransferase activity using GST-SETDB1 K867, which is the K867 mutant of the GST fusion full-length SETDB1 protein (WT), and the activity of GST-SETDB1 K867 almost disappeared (S3 Fig). These results show that SETDB1 ubiquitination at lysine 867 is necessary for full H3K9 methyltransferase activity. In addition, we investigated whether the catalytically inactive SETDB1 mutants were modified by ubiquitin. Schultz *et al*. reported that the H1224K, C1226A, and C1279Y mutants of SETDB1 showed impaired H3K9 methyltransferase activity [7]. We introduced a mutated version of each of these three amino acid residues into the DEL570 protein. These proteins were purified by affinity-chromatography from HeLa cell extracts and were subjected to immunoblot analysis with an anti-ubiquitin antibody (S4 Fig). We detected a band of ubiquitinated protein from all of the mutated constructs. These results indicate that the catalytically inactive SETDB1 mutants did not create the methyltransferase activity by ubiquitination of K867.

### SETDB1 Ubiquitination at Lysine 867 Controls the Expression of the SETDB1 Target Gene

**Fig 6:**
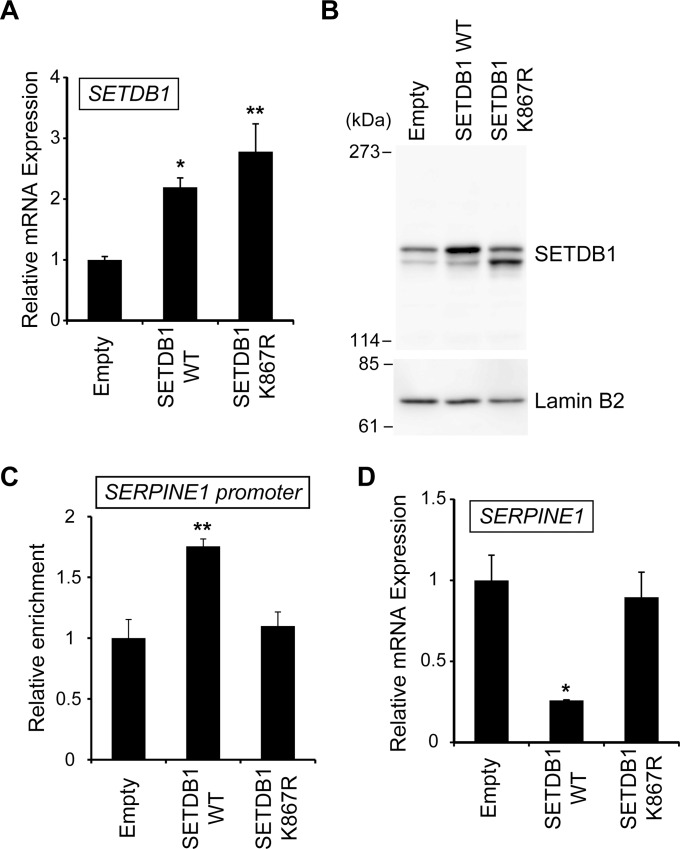
SETDB1 target gene expression is regulated by SETDB1 ubiquitination. HeLa cells were transfected with pcDNA3-hSETDB1 (SETDB1 WT), pcDNA3-hSETDB1 K867R (SETDB1 K867R), or pcDNA3 (Empty). (A and D) mRNA levels were normalized to those of cyclophilin A mRNA. All values are expressed as the mean ± SEM (n = 3). *P < 0.05 or **P < 0.01 compared with the value of Empty. (B) Whole cell extracts were prepared and aliquots [30 μg (for SETDB1) or 10 μg (for lamin B2) protein/lane] were subjected to SDS-PAGE. Immunoblots were probed with anti-SETDB1 antibody or anti-lamin B2 antibody. (C) Purified genomic DNA after ChIP was analyzed by real-time PCR. Data was normalized to the input DNA. Fold change was calculated relative to Empty. All values are expressed as the mean ± SEM (n = 3). **P < 0.01 compared with the value of Empty.

SETDB1 suppresses the expression of its target genes by methylating H3K9 [7, 8, 23]. Since, SETDB1 ubiquitination was found to be important for the H3K9 methyltransferase activity as seen in Fig 5B, we predicted that this ubiquitination was involved in the change in the expression of the SETDB1 target gene. First, we transfected a full-length SETDB1 expression vector (SETDB1 WT), a full-length SETDB1 K867R expression vector (SETDB1 K867R), or a pcDNA3 vector (Empty) into HeLa cells and detected SETDB1 mRNA levels by quantitative RT-PCR. The results showed that SETDB1 mRNA was significantly increased in HeLa cells transfected with SETDB1 WT or SETDB1 K867R as compared to the cells transfected with Empty (Fig 6A). We also examined the protein levels of SETDB1 in a western blotting experiment by using anti-SETDB1 antibody. As shown in Fig 6B, the upper band derived from the ubiquitinated SETDB1 protein was increased in cells transfected with SETDB1 WT as compared to the cells transfected with Empty. In the case of cells transfected with SETDB1 K867R, the lower band derived from the unmodified SETDB1 protein was increased as compared to the cells transfected with Empty.

We next investigated the direct effect of SETDB1 using ChIP assays with an anti-H3K9me3 antibody in HeLa cells. ChIP analysis showed that H3K9me3 level was significantly higher in the promoter region of the *SERPINE1 gene*, a SETDB1 target gene, after transfection with SETDB1 WT, whereas it did not change in the cells transfected with SETDB1 K867R (Fig 6C) [24]. These changes were not observed in control experiments using a negative control genomic region (S5A Fig). Finally, quantitative RT-PCR was performed to analyze the effect of SETDB1 ubiquitination on the expression of *SERPINE1*. The results showed that *SERPINE1* mRNA level was reduced to approximately 0.3-fold after cell transfection with SETDB1 WT, whereas it did not change in the cells transfected with SETDB1 K867R (Fig 6D). In parallel, we examined the mRNA levels of other H3K9 enzyme target genes (S5B Fig). The expression level of *KLF11*, which is the SUV39H1 target gene [25], was unchanged by SETDB1 ubiquitination. Consistent with the results for *KLF11* mRNA, the *MAGEA5* and *MAGEA12* mRNA levels, transcribed from the Mage-A gene family that is targeted by G9a and GLP [26, 27], were unchanged by SETDB1 ubiquitination. These results suggest that the ubiquitination of SETDB1 at lysine 867 controls the expression of its target gene by activating its H3K9 methyltransferase activity.

## Discussion

In this study, we found that SETDB1 (570–1291) is modified by ubiquitination in HeLa and Sf9 cells (Fig 3). We also found that the ubiquitination upregulates its H3K9 methyltransferase activity (Fig 5). Ubiquitination is the process by which a 76 amino acid ubiquitin molecule is covalently attached to a lysine residue of a target protein [28, 29]. Different types of ubiquitination are distinguished by the number of ubiquitin moieties conjugated to the protein. In most instances, poly-ubiquitination targets proteins for degradation by the 26S proteasome and controls many cellular processes [30, 31], whereas mono-, multiple mono-, and oligoubiquitination have nondegradative functions, such as protein trafficking, kinase activation, and DNA damage repair [32, 33]. These observations raise the possibility that the ubiquitination of SETDB1 plays a nondegradative role as observed with mono-, multiple mono-, and oligoubiquitination of proteins.

We revealed that the ubiquitination of K867 of the human SETDB1 protein upregulates its H3K9 methyltransferase activity. SETDB1 protein expressed in *Escherichia coli* that did not undergo PTMs failed to show the enzyme activity [7]. However, SETDB1 protein expressed in insect and mammalian cells that underwent PTMs exhibited the enzyme activity. These reports support our findings that the ubiquitination of SETDB1 is necessary for the enzyme activity. In contrast, K867R still retained approximately 30% of the methyltransferase activity observed for DEL570 (Fig 5B). DEL570 does not have an N-terminal region; therefore, we assume that the remaining parts of SETDB1 may form the enzymatic active structure without ubiquitination. Indeed, the methyltransferase activity of GST-SETDB1 K867 which is the K867 mutant of the GST fusion full-length SETDB1 protein (WT) was almost disappeared (S3 Fig).

**Fig 7:**
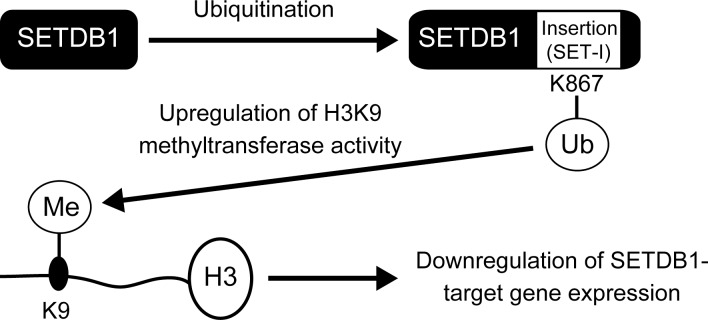
Schematic model of the SETDB1 ubiquitination and H3K9 methyltransferase activity described in this study.

We compared the lysine residue at amino acid position 867 of human SETDB1 with that of other species (S6 Fig). The K867 residue is highly conserved among mammals (*Macaca mulatta*, *Mus musculus*, and *Rattus norvegicus*), amphibia (*Xenopus laevis*), fishes (*Danio rerio*), and insects (*Drosophila melanogaster*). In addition, this lysine site is located within an insert region (SET-I) where the SET domain is bifurcated. SET-I is approximately 30 amino acids in length and is a region of considerable sequence variability within the SET family. Although the lengths of the SET-I region vary across family members, the structural architecture of SET-I is conserved and it is structurally static [34, 35]. In the ternary structure of the H4K20 methyltransferase Pr-Set7, SET-I consists of a helix(α-2)-loop-strand(β-5)-loop-strand(β-6) [36]. In case of the structural superimposition of the ternary H3K9 methyltransferase GLP structure with H3K9 methyltransferase G9a, or H3K9 methyltransferase Suv39H2, SET-I is composed of a helix followed by a two-stranded anti-parallel β-sheet, linked by loops of variable lengths [37]. Based on its relatively rigidity and structural conservation, the SET-I region among different SET domain proteins contributes to the mechanism of substrate recognition. Human SETDB1 has a 363 amino acid insertion between amino acids 846 and 1208, and it is predicted to display more sequence and structural variability in this long SET-I region [35]. We speculate that the ubiquitination of SET-I facilitates the adoption of a stable three-dimensional structure that promotes H3K9 methyltransferase activity (Fig 7). The promotion of H3K9 methylation suppresses the expression of its target genes. Further investigations are needed to elucidate how the ubiquitination of SETDB1 influences its structure and enzymatic activity.

In this study, we showed that the region between amino acids 570 and 680 is essential for the ubiquitination of K867 in human SETDB1 (Fig 3). An MBD (from 594 to 665) is located within this region of SETDB1; however, little is known about its function. In general, the MBD is known to bind to methylated CpGs, and recruit a variety of chromatin remodeling proteins such as histone deacetylase (HDAC) [38, 39]. The MBD of human SETDB1 contains two conserved DNA-interacting arginine residues (R615 and R636) [40], that are reported to facilitate direct contact between DNA and the components of the MBD like MBD1 and MeCP2 [41, 42]. We investigated the effect of ubiquitination on the two arginine residues that influence the DNA-binding ability of MBD, and we found that SETDB1 ubiquitination is not affected by the substitution of these arginines to alanines (S7 Fig). On the other hand, MBD components including MBD3 and MeCP2 are known to mediate protein-protein interactions [43, 44]. Therefore, the MBD of SETDB1 also has the potential to mediate interactions with other proteins, such as E3 ubiquitin ligases, which may be involved in the ubiquitination of SETDB1. Further investigations are required to clarify the mechanisms of SETDB1 ubiquitination that are mediated by its MBD.

In conclusion, we have demonstrated for the first time that ubiquitination of human SETDB1 at lysine 867 is required for its H3K9 methyltransferase activity and regulation of the expression of its target gene. Since, SETDB1 controls a variety of intracellular physiologic processes through its H3K9 methyltransferase activity, this finding will be useful in planning future studies to elucidate its functions.

## Supporting Information

S1 Fig The PTMs of SETDB1 are associated with its H3K9 methyltransferase activity in Sf9 cells. (A) GST affinity-purified SETDB1 proteins in Sf9 cells were resolved on 7% SDS-PAGE. Western blot analysis was performed using anti-GST antibody. Asterisks indicate degradation products from SETDB1. (B) H3K9 methyltransferase activity of GST affinity-purified SETDB1 proteins in Sf9 cells was measured. The values represent means±SEM (n = 3). N. D.: not detected.
(TIF)

S2 Fig Amino acid sequence of human SETDB1 (570–1291). The red text denotes the amino acid residues that were detected by LC-MS/MS analysis; the black box denotes the region between amino acids 845 and 886; the white text on a black background denotes invariant residues.
(TIF)

S3 Fig Ubiquitination of K867 of GST-SETDB1 WT upregulates its H3K9 methyltransferase activity in HeLa cells. SETDB1 proteins were expressed as GST fusion proteins in HeLa cells and purified on glutathione-sepharose beads. The H3K9 methyltransferase activity of the GST affinity-purified SETDB1 proteins in HeLa cells was measured. The values represent mean ± SEM (n = 3).
(TIF)

S4 Fig The catalytically inactive SETDB1 is still ubiquitinated in HeLa cells. (A) Schematic view of the mutation map for GST-SETDB1 (570–1291). (B and C) SETDB1 proteins were expressed as GST fusion proteins in HeLa cells and purified on glutathione-sepharose beads. The purified SETDB1 proteins were resolved on 5% SDS-PAGE and electroblotted onto PVDF membranes. Western blots of GST-SETDB1 proteins were probed with anti-SETDB1 antibody or anti-ubiquitin antibody.
(TIF)

S5 Fig Negative control gene is not regulated by SETDB1 ubiquitination. HeLa cells were transfected with pcDNA3-hSETDB1 (SETDB1 WT), pcDNA3-hSETDB1 K867R (SETDB1 K867R), or pcDNA3 (Empty). (A) Purified genomic DNA after ChIP was analyzed by real-time PCR. Data were normalized to the input DNA. Fold change was calculated relative to Empty. All values are expressed as the mean ± SEM (n = 3). (B) mRNA levels were normalized to those of cyclophilin A mRNA. All values are expressed as the mean ± SEM (n = 3).
(TIF)

S6 Fig A protein sequence alignment of human SETDB1 between amino acids 857 and 877. An alignment of the SETDB1 protein from *Homo sapiens*, *Macaca mulatta*, *Mus musculus*, *Rattus norvegicus*, *Xenopus laevis*, *Danio rerio*, and *Drosophila melanogaster* is shown. The white text on a black background denotes invariant residues; the black text on a gray background indicates conserved residues.
(TIF)

S7 Fig Two conserved DNA-interacting arginine residues in the methyl-CpG-binding domain (MBD) are not influenced by PTMs in HeLa cells. (A) Schematic view of the mutation map for GST-SETDB1 (570–1291). (B) Twenty-four hours after transfection, cell extracts were prepared, and the extracts were subjected to SDS-PAGE. Western blot analyses were probed with anti-SETDB1 antibody or anti-lamin B2 antibody.
(TIF)

S1 Table List of proteotypic peptides from SETDB1 (570–1291). (XLSX)

## References

[1] Farley AR, Link AJ. Identification and quantification of protein posttranslational modifications. Methods Enzymol. 2009;463:725–63. 10.1016/S0076-6879(09)63040-8 . 19892200

[2] Karve TM, Cheema AK. Small changes huge impact: the role of protein posttranslational modifications in cellular homeostasis and disease. J Amino Acids. 2011;2011:207691 10.4061/2011/207691 22312457 PMC3268018

[3] Chu CS, Lo PW, Yeh YH, Hsu PH, Peng SH, Teng YC, et al O-GlcNAcylation regulates EZH2 protein stability and function. Proc Natl Acad Sci U S A. 2014;111(4):1355–60. 10.1073/pnas.1323226111 24474760 PMC3910655

[4] Watson GW, Wickramasekara S, Palomera-Sanchez Z, Black C, Maier CS, Williams DE, et al SUV39H1/H3K9me3 attenuates sulforaphane-induced apoptotic signaling in PC3 prostate cancer cells. Oncogenesis. 2014;3:e131 10.1038/oncsis.2014.47 25486523 PMC4275561

[5] Sampath SC, Marazzi I, Yap KL, Krutchinsky AN, Mecklenbräuker I, Viale A, et al Methylation of a histone mimic within the histone methyltransferase G9a regulates protein complex assembly. Mol Cell. 2007;27(4):596–608. 10.1016/j.molcel.2007.06.026 . 17707231

[6] Rea S, Eisenhaber F, O'Carroll D, Strahl BD, Sun ZW, Schmid M, et al Regulation of chromatin structure by site-specific histone H3 methyltransferases. Nature. 2000;406(6796):593–9. 10.1038/35020506 . 10949293

[7] Schultz DC, Ayyanathan K, Negorev D, Maul GG, Rauscher FJ. SETDB1: a novel KAP-1-associated histone H3, lysine 9-specific methyltransferase that contributes to HP1-mediated silencing of euchromatic genes by KRAB zinc-finger proteins. Genes Dev. 2002;16(8):919–32. 10.1101/gad.973302 11959841 PMC152359

[8] Li H, Rauch T, Chen ZX, Szabó PE, Riggs AD, Pfeifer GP. The histone methyltransferase SETDB1 and the DNA methyltransferase DNMT3A interact directly and localize to promoters silenced in cancer cells. J Biol Chem. 2006;281(28):19489–500. 10.1074/jbc.M513249200 . 16682412

[9] Ceol CJ, Houvras Y, Jane-Valbuena J, Bilodeau S, Orlando DA, Battisti V, et al The histone methyltransferase SETDB1 is recurrently amplified in melanoma and accelerates its onset. Nature. 2011;471(7339):513–7. 10.1038/nature09806 21430779 PMC3348545

[10] Rodriguez-Paredes M, Martinez de Paz A, Simó-Riudalbas L, Sayols S, Moutinho C, Moran S, et al Gene amplification of the histone methyltransferase SETDB1 contributes to human lung tumorigenesis. Oncogene. 2014;33(21):2807–13. 10.1038/onc.2013.239 23770855 PMC4031636

[11] Fei Q, Shang K, Zhang J, Chuai S, Kong D, Zhou T, et al Histone methyltransferase SETDB1 regulates liver cancer cell growth through methylation of p53. Nat Commun. 2015;6:8651 10.1038/ncomms9651 . 26471002 PMC5426523

[12] Kim HA, Koo BK, Cho JH, Kim YY, Seong J, Chang HJ, et al Notch1 counteracts WNT/β-catenin signaling through chromatin modification in colorectal cancer. J Clin Invest. 2012;122(9):3248–59. 10.1172/JCI61216 22863622 PMC3428081

[13] Matsui T, Leung D, Miyashita H, Maksakova IA, Miyachi H, Kimura H, et al Proviral silencing in embryonic stem cells requires the histone methyltransferase ESET. Nature. 2010;464(7290):927–31. 10.1038/nature08858 . 20164836

[14] Wakabayashi K, Okamura M, Tsutsumi S, Nishikawa NS, Tanaka T, Sakakibara I, et al The peroxisome proliferator-activated receptor gamma/retinoid X receptor alpha heterodimer targets the histone modification enzyme PR-Set7/Setd8 gene and regulates adipogenesis through a positive feedback loop. Mol Cell Biol. 2009;29(13):3544–55. 10.1128/MCB.01856-08 19414603 PMC2698772

[15] Bühler D, Raker V, Lührmann R, Fischer U. Essential role for the tudor domain of SMN in spliceosomal U snRNP assembly: implications for spinal muscular atrophy. Hum Mol Genet. 1999;8(13):2351–7. . 10556282 10.1093/hmg/8.13.2351

[16] Pek JW, Anand A, Kai T. Tudor domain proteins in development. Development. 2012;139(13):2255–66. 10.1242/dev.073304 . 22669818

[17] Tachibana K, Gotoh E, Kawamata N, Ishimoto K, Uchihara Y, Iwanari H, et al Analysis of the subcellular localization of the human histone methyltransferase SETDB1. Biochem Biophys Res Commun. 2015;465(4):725–31. 10.1016/j.bbrc.2015.08.065 . 26296461

[18] Ishimoto K, Tachibana K, Hanano I, Yamasaki D, Nakamura H, Kawai M, et al Sterol-regulatory-element-binding protein 2 and nuclear factor Y control human farnesyl diphosphate synthase expression and affect cell proliferation in hepatoblastoma cells. Biochem J. 2010;429(2):347–57. 10.1042/BJ20091511 . 20450493

[19] Ishimoto K, Nakamura H, Tachibana K, Yamasaki D, Ota A, Hirano K, et al Sterol-mediated regulation of human lipin 1 gene expression in hepatoblastoma cells. J Biol Chem. 2009;284(33):22195–205. 10.1074/jbc.M109.028753 19553673 PMC2755944

[20] Tachibana K, Kobayashi Y, Tanaka T, Tagami M, Sugiyama A, Katayama T, et al Gene expression profiling of potential peroxisome proliferator-activated receptor (PPAR) target genes in human hepatoblastoma cell lines inducibly expressing different PPAR isoforms. Nucl Recept. 2005;3:3 10.1186/1478-1336-3-3 16197558 PMC1262764

[21] Marotti LA, Newitt R, Wang Y, Aebersold R, Dohlman HG. Direct identification of a G protein ubiquitination site by mass spectrometry. Biochemistry. 2002;41(16):5067–74. . 11955054 10.1021/bi015940q

[22] Peng J, Schwartz D, Elias JE, Thoreen CC, Cheng D, Marsischky G, et al A proteomics approach to understanding protein ubiquitination. Nat Biotechnol. 2003;21(8):921–6. 10.1038/nbt849 . 12872131

[23] Sarraf SA, Stancheva I. Methyl-CpG binding protein MBD1 couples histone H3 methylation at lysine 9 by SETDB1 to DNA replication and chromatin assembly. Mol Cell. 2004;15(4):595–605. 10.1016/j.molcel.2004.06.043 . 15327775

[24] Karimi MM, Goyal P, Maksakova IA, Bilenky M, Leung D, Tang JX, et al DNA methylation and SETDB1/H3K9me3 regulate predominantly distinct sets of genes, retroelements, and chimeric transcripts in mESCs. Cell Stem Cell. 2011;8(6):676–87. 10.1016/j.stem.2011.04.004 21624812 PMC3857791

[25] Lomberk G, Mathison AJ, Grzenda A, Seo S, DeMars CJ, Rizvi S, et al Sequence-specific recruitment of heterochromatin protein 1 via interaction with Krüppel-like factor 11, a human transcription factor involved in tumor suppression and metabolic diseases. J Biol Chem. 2012;287(16):13026–39. 10.1074/jbc.M112.342634 22318730 PMC3339955

[26] Tachibana M, Sugimoto K, Nozaki M, Ueda J, Ohta T, Ohki M, et al G9a histone methyltransferase plays a dominant role in euchromatic histone H3 lysine 9 methylation and is essential for early embryogenesis. Genes Dev. 2002;16(14):1779–91. 10.1101/gad.989402 12130538 PMC186403

[27] Tachibana M, Ueda J, Fukuda M, Takeda N, Ohta T, Iwanari H, et al Histone methyltransferases G9a and GLP form heteromeric complexes and are both crucial for methylation of euchromatin at H3-K9. Genes Dev. 2005;19(7):815–26. 10.1101/gad.1284005 15774718 PMC1074319

[28] Pickart CM. Mechanisms underlying ubiquitination. Annu Rev Biochem. 2001;70:503–33. 10.1146/annurev.biochem.70.1.503 . 11395416

[29] Pickart CM, Eddins MJ. Ubiquitin: structures, functions, mechanisms. Biochim Biophys Acta. 2004;1695(1–3):55–72. 10.1016/j.bbamcr.2004.09.019 . 15571809

[30] Glickman MH, Ciechanover A. The ubiquitin-proteasome proteolytic pathway: destruction for the sake of construction. Physiol Rev. 2002;82(2):373–428. 10.1152/physrev.00027.2001 . 11917093

[31] Wolf DH, Hilt W. The proteasome: a proteolytic nanomachine of cell regulation and waste disposal. Biochim Biophys Acta. 2004;1695(1–3):19–31. 10.1016/j.bbamcr.2004.10.007 . 15571806

[32] Schnell JD, Hicke L. Non-traditional functions of ubiquitin and ubiquitin-binding proteins. J Biol Chem. 2003;278(38):35857–60. 10.1074/jbc.R300018200 . 12860974

[33] Mukhopadhyay D, Riezman H. Proteasome-independent functions of ubiquitin in endocytosis and signaling. Science. 2007;315(5809):201–5. 10.1126/science.1127085 . 17218518

[34] Xiao B, Wilson JR, Gamblin SJ. SET domains and histone methylation. Curr Opin Struct Biol. 2003;13(6):699–705. . 14675547 10.1016/j.sbi.2003.10.003

[35] Marmorstein R. Structure of SET domain proteins: a new twist on histone methylation. Trends Biochem Sci. 2003;28(2):59–62. 10.1016/S0968-0004(03)00007-0 . 12575990

[36] Xiao B, Jing C, Kelly G, Walker PA, Muskett FW, Frenkiel TA, et al Specificity and mechanism of the histone methyltransferase Pr-Set7. Genes Dev. 2005;19(12):1444–54. 10.1101/gad.1315905 15933069 PMC1151661

[37] Wu H, Min J, Lunin VV, Antoshenko T, Dombrovski L, Zeng H, et al Structural biology of human H3K9 methyltransferases. PLoS One. 2010;5(1):e8570 10.1371/journal.pone.0008570 20084102 PMC2797608

[38] Roloff TC, Ropers HH, Nuber UA. Comparative study of methyl-CpG-binding domain proteins. BMC Genomics. 2003;4(1):1 10.1186/1471-2164-4-1 12529184 PMC149351

[39] Baubec T, Ivánek R, Lienert F, Schübeler D. Methylation-dependent and -independent genomic targeting principles of the MBD protein family. Cell. 2013;153(2):480–92. 10.1016/j.cell.2013.03.011 . 23582333

[40] Hashimoto H, Vertino PM, Cheng X. Molecular coupling of DNA methylation and histone methylation. Epigenomics. 2010;2(5):657–69. 10.2217/epi.10.44 21339843 PMC3039846

[41] Ohki I, Shimotake N, Fujita N, Jee J, Ikegami T, Nakao M, et al Solution structure of the methyl-CpG binding domain of human MBD1 in complex with methylated DNA. Cell. 2001;105(4):487–97. . 11371345 10.1016/s0092-8674(01)00324-5

[42] Ho KL, McNae IW, Schmiedeberg L, Klose RJ, Bird AP, Walkinshaw MD. MeCP2 binding to DNA depends upon hydration at methyl-CpG. Mol Cell. 2008;29(4):525–31. 10.1016/j.molcel.2007.12.028 . 18313390

[43] Saito M, Ishikawa F. The mCpG-binding domain of human MBD3 does not bind to mCpG but interacts with NuRD/Mi2 components HDAC1 and MTA2. J Biol Chem. 2002;277(38):35434–9. 10.1074/jbc.M203455200 . 12124384

[44] Carro S, Bergo A, Mengoni M, Bachi A, Badaracco G, Kilstrup-Nielsen C, et al A novel protein, Xenopus p20, influences the stability of MeCP2 through direct interaction. J Biol Chem. 2004;279(24):25623–31. 10.1074/jbc.M402571200 . 15056664

