# Supplementary figures and images for: Ubiquitination of Lysine 867 of the Human SETDB1 Protein Upregulates Its Histone H3 Lysine 9 (H3K9) Methyltransferase Activity
Source: PLoS One. 2016 Oct 31;11(10):e0165766. doi: 10.1371/journal.pone.0165766 (PMC5087952; doi:10.1371/journal.pone.0165766)

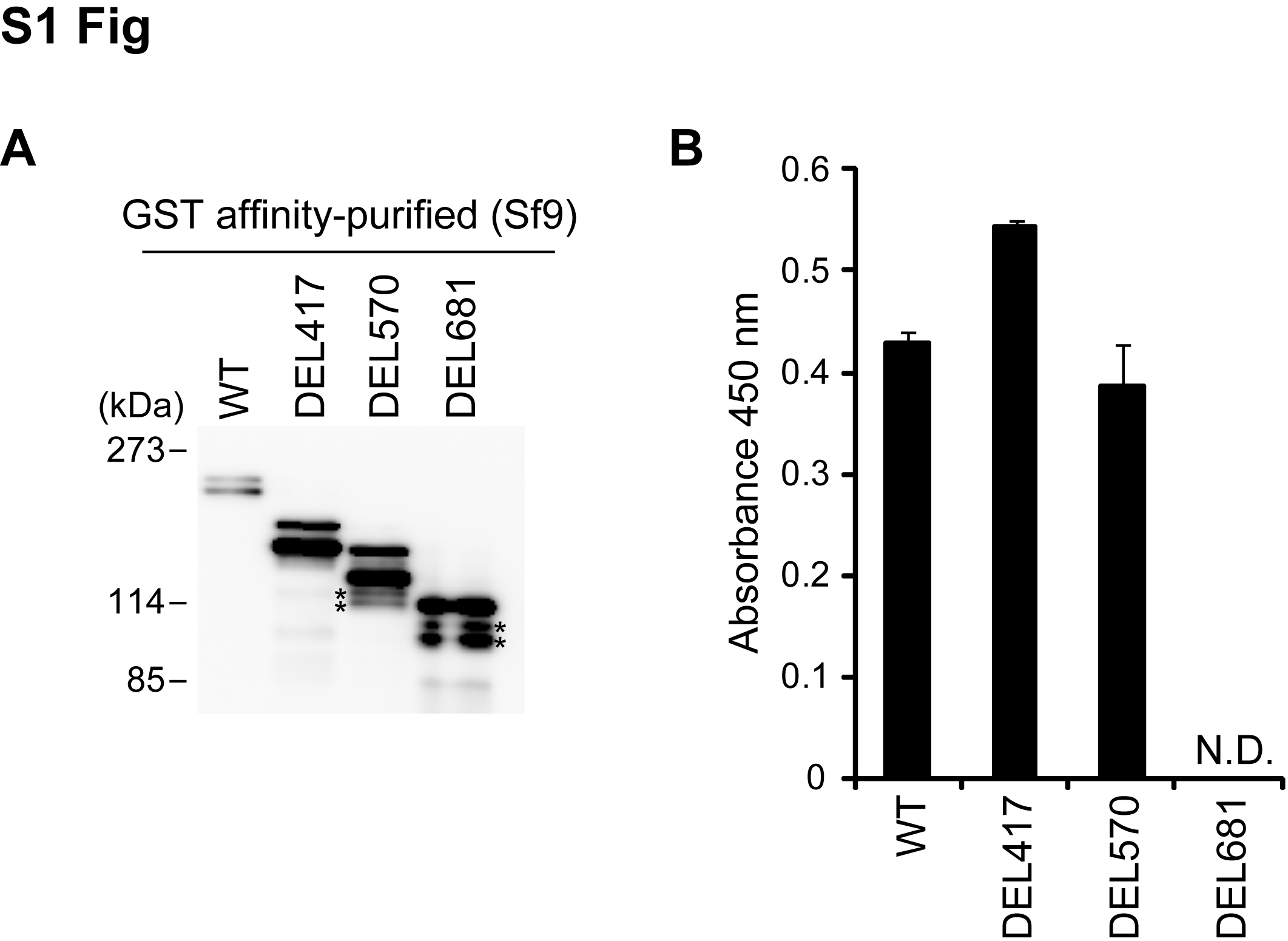

Supplement: S1 Fig — (A) GST affinity-purified SETDB1 proteins in Sf9 cells were resolved on 7% SDS-PAGE. Western blot analysis was performed using anti-GST antibody. Asterisks indicate degradation products from SETDB1. (B) H3K9 methyltransferase activity of GST affinity-purified SETDB1 proteins in Sf9 cells was measured. The values represent means±SEM (n = 3). N. D.: not detected. (TIF) [file pone.0165766.s001.tif]

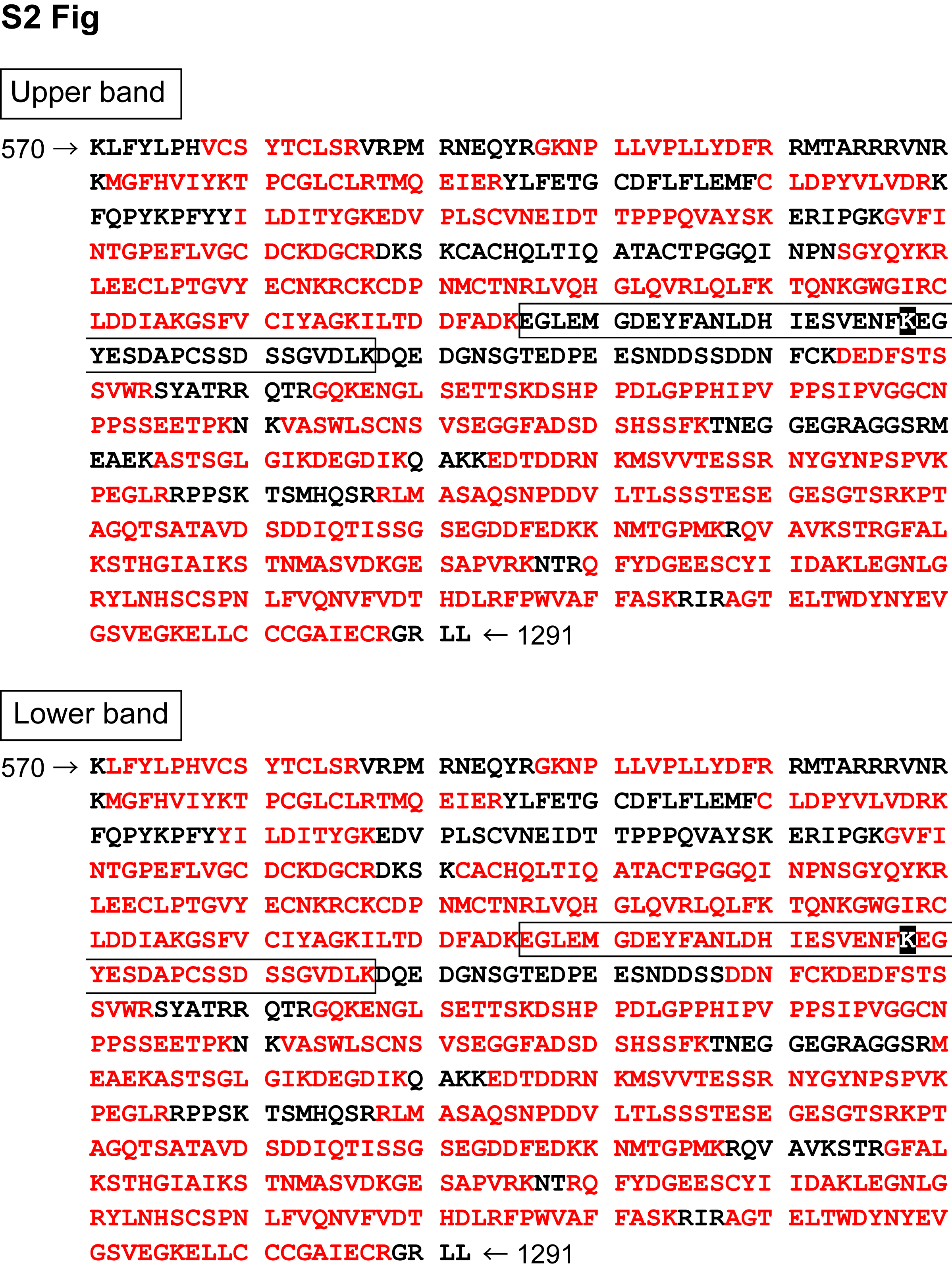

Supplement: S2 Fig — The red text denotes the amino acid residues that were detected by LC-MS/MS analysis; the black box denotes the region between amino acids 845 and 886; the white text on a black background denotes invariant residues. (TIF) [file pone.0165766.s002.tif]

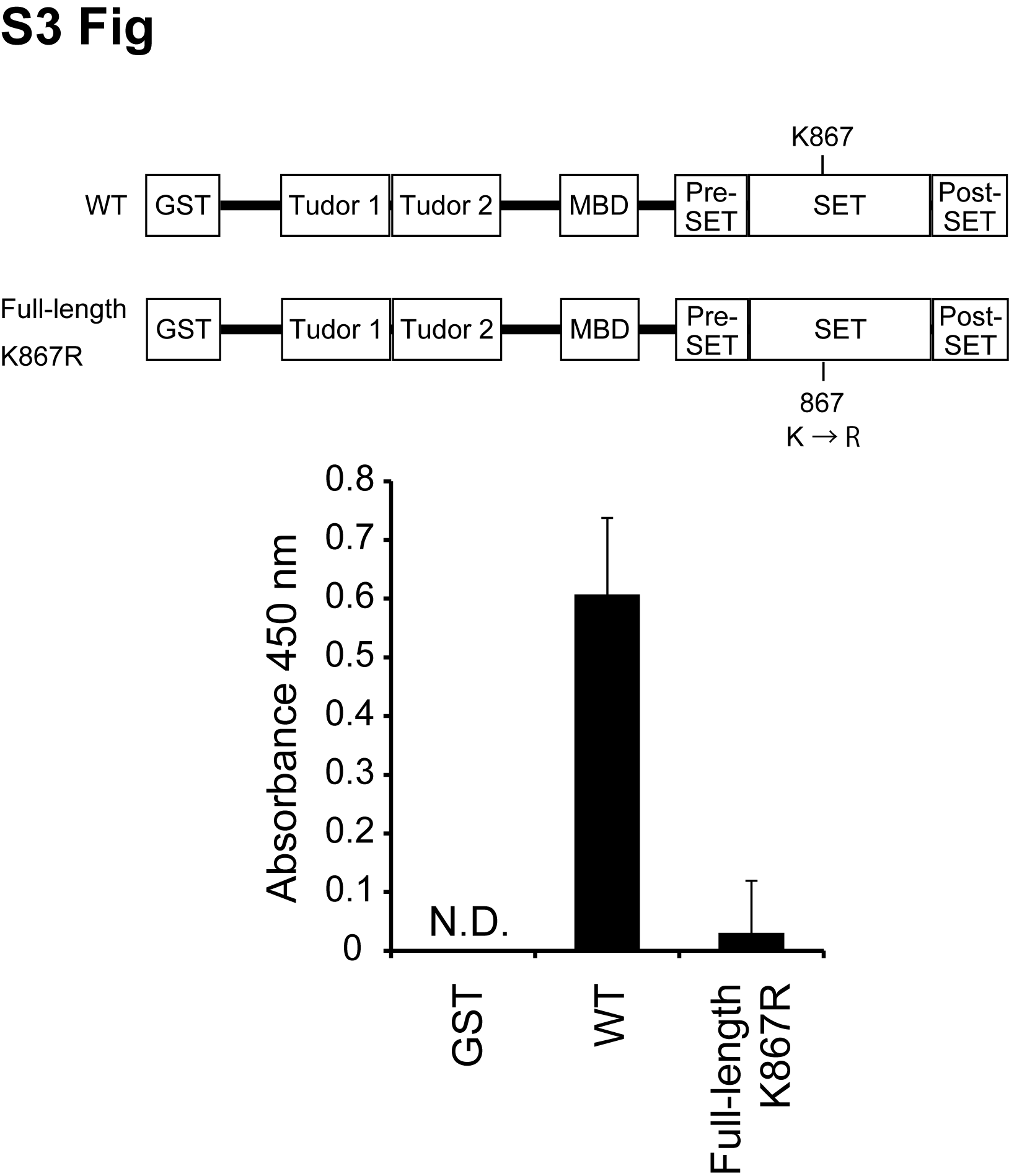

Supplement: S3 Fig — SETDB1 proteins were expressed as GST fusion proteins in HeLa cells and purified on glutathione-sepharose beads. The H3K9 methyltransferase activity of the GST affinity-purified SETDB1 proteins in HeLa cells was measured. The values represent mean ± SEM (n = 3). (TIF) [file pone.0165766.s003.tif]

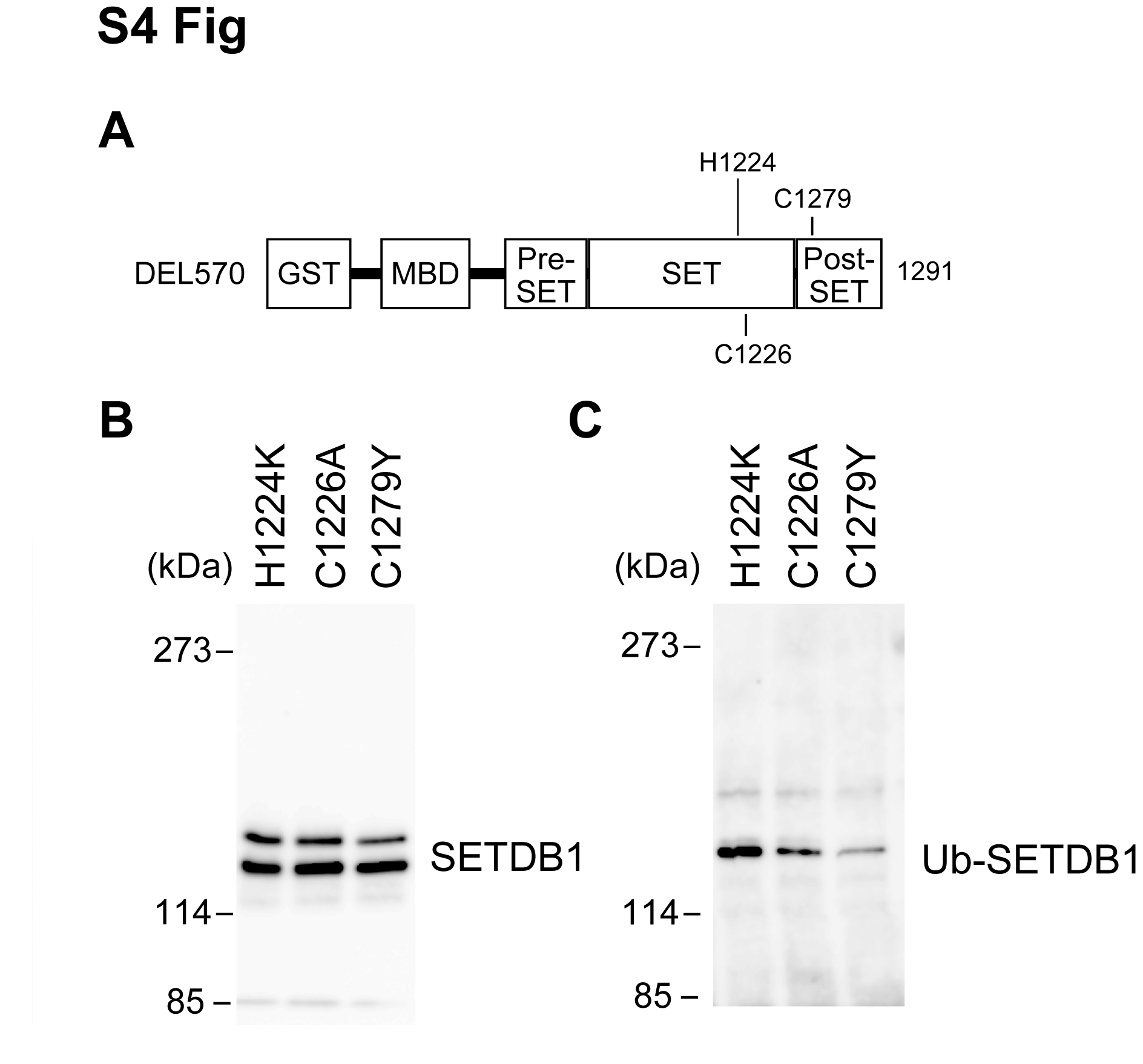

Supplement: S4 Fig — (A) Schematic view of the mutation map for GST-SETDB1 (570–1291). (B and C) SETDB1 proteins were expressed as GST fusion proteins in HeLa cells and purified on glutathione-sepharose beads. The purified SETDB1 proteins were resolved on 5% SDS-PAGE and electroblotted onto PVDF membranes. Western blots of GST-SETDB1 proteins were probed with anti-SETDB1 antibody or anti-ubiquitin antibody. (TIF) [file pone.0165766.s004.tif]

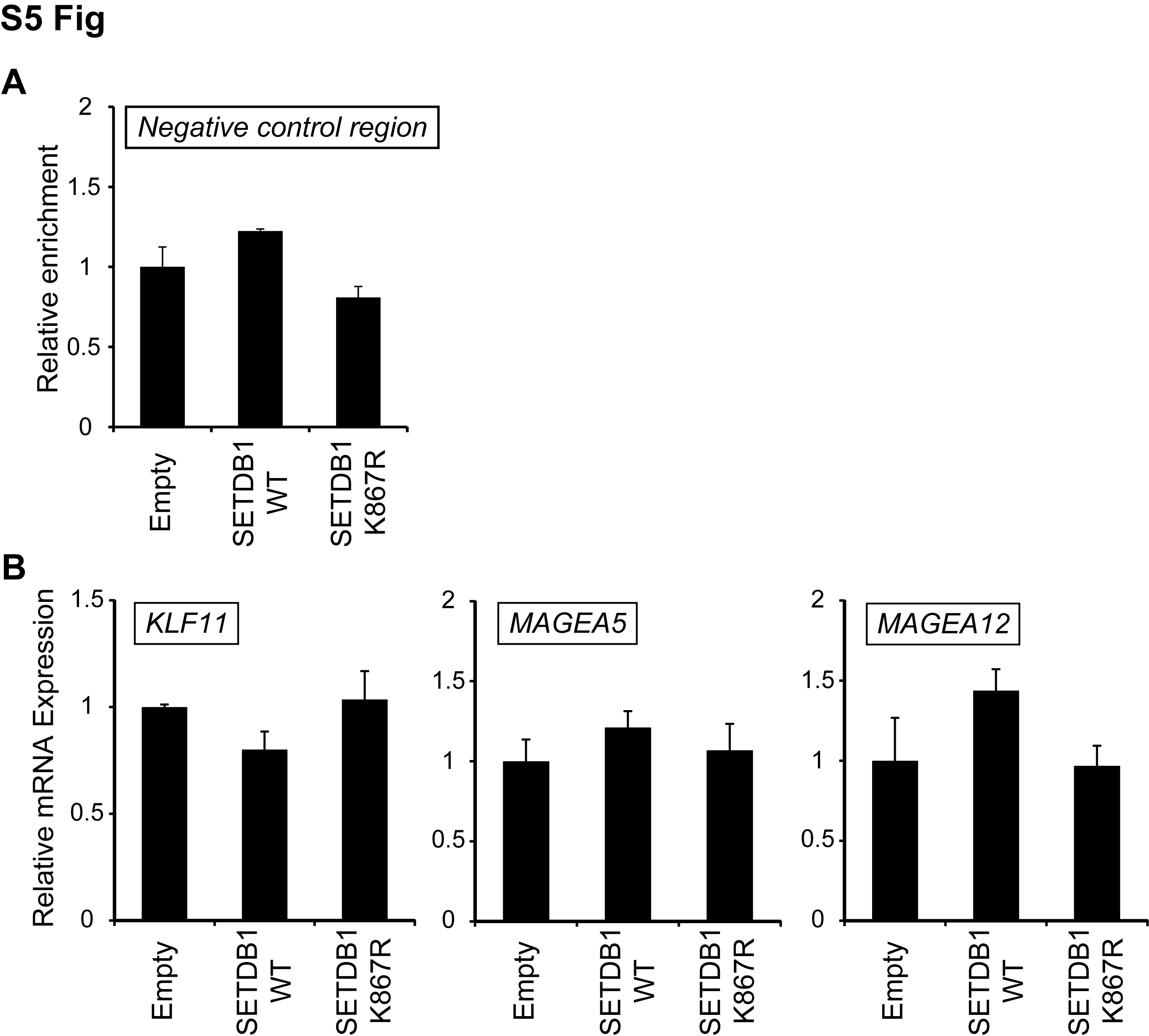

Supplement: S5 Fig — HeLa cells were transfected with pcDNA3-hSETDB1 (SETDB1 WT), pcDNA3-hSETDB1 K867R (SETDB1 K867R), or pcDNA3 (Empty). (A) Purified genomic DNA after ChIP was analyzed by real-time PCR. Data were normalized to the input DNA. Fold change was calculated relative to Empty. All values are expressed as the mean ± SEM (n = 3). (B) mRNA levels were normalized to those of cyclophilin A mRNA. All values are expressed as the mean ± SEM (n = 3). (TIF) [file pone.0165766.s005.tif]

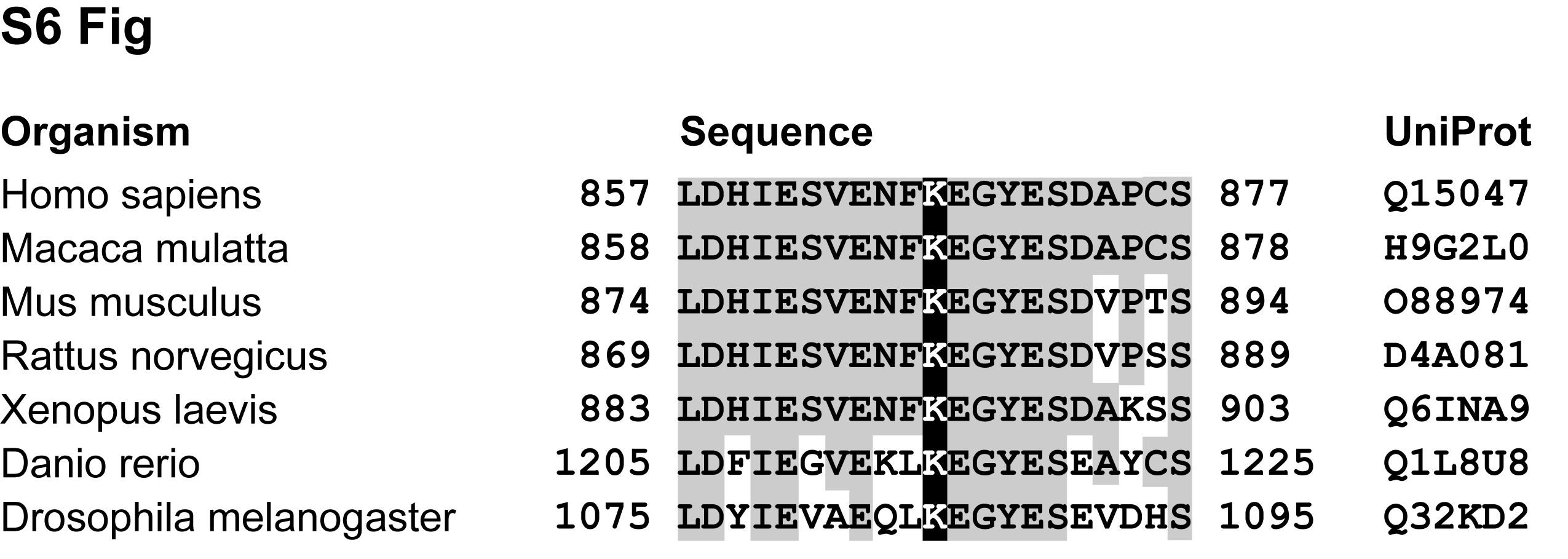

Supplement: S6 Fig — An alignment of the SETDB1 protein from Homo sapiens, Macaca mulatta, Mus musculus, Rattus norvegicus, Xenopus laevis, Danio rerio, and Drosophila melanogaster is shown. The white text on a black background denotes invariant residues; the black text on a gray background indicates conserved residues. (TIF) [file pone.0165766.s006.tif]

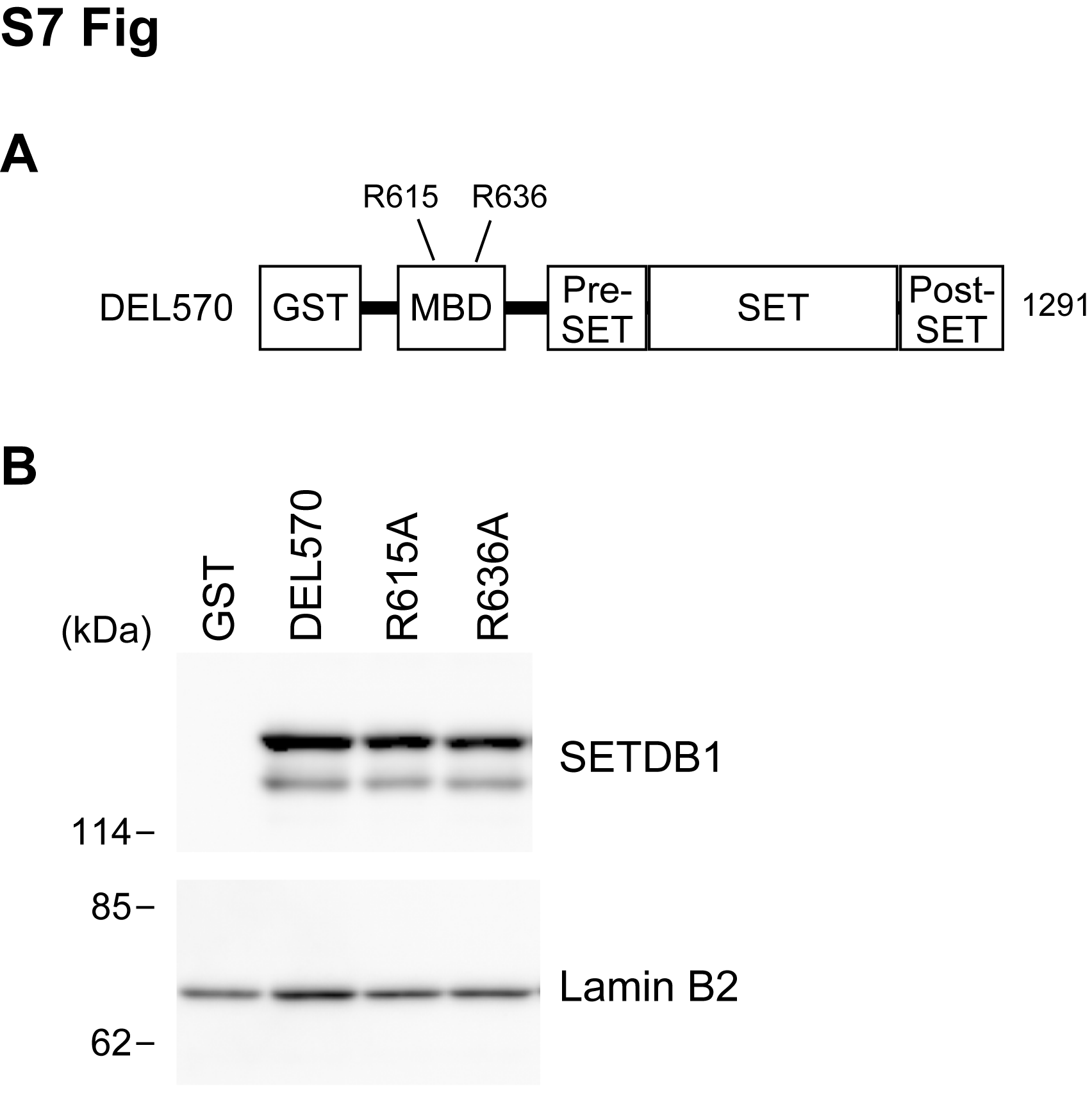

Supplement: S7 Fig — (A) Schematic view of the mutation map for GST-SETDB1 (570–1291). (B) Twenty-four hours after transfection, cell extracts were prepared, and the extracts were subjected to SDS-PAGE. Western blot analyses were probed with anti-SETDB1 antibody or anti-lamin B2 antibody. (TIF) [file pone.0165766.s007.tif]
